# Impact of thermoneutral acclimation on a murine model of polymicrobial peritonitis

**DOI:** 10.1371/journal.pone.0322855

**Published:** 2025-05-30

**Authors:** Goldia Chan, Christopher Fry, Jean Nemzek

**Affiliations:** Unit for Laboratory Animal Medicine, University of Michigan, Ann Arbor, Michigan, United States of America; Medical School, University of Pecs, HUNGARY

## Abstract

To examine the effects of ambient temperature on the reproducibility and translation of a murine sepsis model, we hypothesized that acclimation of mice in temperatures within their thermoneutral zone would alter immune responses and outcomes compared to standard housing temperatures. Mice housed for one week in thermoneutral (30°C) as compared to standard (22°C) conditions displayed lower counts of circulating neutrophils (0.52 ± 0.20 vs. 1.10 ± 0.54 x10^3^/μL; p = 0.011) and peritoneal macrophages (0.80 ± 0.57 vs. 1.62 ± 0.62 x 10^5^/μL; p = 0.002) as well as reduced *in vitro* production of IFN-γ by stimulated splenocytes (0.38 ± 0.68 vs 2.55 ± 0.76 x10^4^ pg/mL, respectively, p = 0.004). After one week of temperature acclimation followed by CLP, the 7-day mortality was significantly lower under thermoneutral as compared to standard temperatures (80% vs 30%, respectively; p = 0.012), although core body temperature was preserved (average for 24 hours: 36.4 ± 1.3°C vs 31.7 ± 4.7°C; p < 0.0001). The lower survival was accompanied by increased systemic IL-6 levels (3.8 ± 3.3 vs 1.9 ± 1.3 x10^3^ pg/mL; p = 0.04) and less robust influx of neutrophils into the peritoneum (1.68 ± 1.07 vs. 4.20 ± 2.46 x10^5^/μL, respectively; p = 0.0003). Overall, thermoneutral temperatures impacted innate immune parameters before and after CLP, producing distinctly different outcomes. Therefore, ambient temperature is an important variable that could impact model reproducibility and should be reported for the acclimation period and experimentation phases of murine sepsis studies.

## Introduction

Sepsis is a multi-factorial syndrome defined as life-threatening organ dysfunction due to dysregulated host response to infection [1]. The global incidence of sepsis is > 19.4 million cases per year and it is a leading cause of death in critical care units [2]. Despite decades of sepsis research, the treatment of sepsis has gained little traction and is plagued by the repeated failure of preclinical studies to translate to the bedside. Consequently, the clinical relevance of preclinical models has undergone a high level of scrutiny. Mouse models, particularly those employing cecal ligation and puncture (CLP), are the most used in sepsis and have some distinct advantages [3]. However, these models have been criticized for their failure to manifest some key pathophysiologic features and/or inability to replicate the heterogeneity of septic humans [4,5]. Several technical modifications [3,6,7] and standardization [8] of rodent models have been suggested to improve reproducibility and translation of preclinical sepsis studies. However, the key to providing more translatable data from murine models of sepsis may lie in the resolution of fundamental differences between species that impact inflammation and immunity.

Due to vast disparity in body size, mice and humans have developed different thermoregulatory mechanisms and existing standards for rodent housing exacerbate the physiologic effects of these differences. Currently, the Guide for the Care and Use of Laboratory Animals recommends room temperatures ranging from 20–26°C [9], temperatures considered comfortable for animal caretakers [10]. However, the thermoneutral zone (TNZ) of mice lies between 26–34°C [9]. Therefore, in standard laboratory settings, mice must expend energy up to 3.1 times basal metabolism to maintain normal body temperature [10]. This chronic cold stress triggers adaptive glucocorticoid production and sympathetic nervous system activation in mice, resulting in secondary immunosuppressive effects [11]. In contrast, mice housed at thermoneutrality display much lower energy expenditure, closer to the physiology of unstressed humans. Correction of cold stress also alters the murine immune response and has shown effects on models of several human inflammatory diseases, including cancer [12], graft-versus-host-disease [13], endotoxin exposure [14,15], and infections [16,17]. In some cases, the effects of thermoneutral housing have produced models that are more representative of the human disease [10,18]. Therefore, it is possible that thermoneutral housing could alter murine sepsis models to yield better translation.

Specific to bacterial sepsis, few studies have examined the effects of ambient temperature on rodent models and many do not report this variable [19]. Our previous work demonstrated that housing mice at 30°C immediately after CLP improved local bacterial clearance and survival compared to housing at 22°C [20]. In that study design, mice were initially acclimated in standard housing conditions and, therefore, were still cold-stressed at the time of CLP. For the study presented here, we theorized that exposure to thermoneutral temperatures prior to induction of sepsis would further alter the inflammatory responses and outcomes in the CLP model of sepsis. Specifically, this study aimed to compare the course of sepsis in mice acclimated to housing temperatures of either 30°C (thermoneutral) or 22°C (standard) for one week prior to CLP and during the development of sepsis. Based on differences in survival, select biomarkers from systemic (plasma) and local (peritoneal) compartments were examined prior to and during early sepsis to further characterize the effects of ambient temperature on the CLP model. The results demonstrated remarkable differences in survival, patterns of thermoregulation, and immune responses with exposure to thermoneutral temperatures for one week prior to induction of sepsis.

## Materials and methods

### Ethical Statement

The animal studies were approved by the Institutional Animal Care and Use Committee of the University of Michigan (Protocol number: PRO00011140) in compliance with the eighth edition of the Guide for the Care and Use of Laboratory Animals [9].

### Animals

Male (6–8 wk) C57BL/6J mice (The Jackson Laboratory, Bar Harbor, ME) were used in all experiments. Upon arrival at out facility, mice were SPF for viruses, bacteria, and parasites including mouse hepatitis virus, minute virus of mice, mouse parvovirus, enzootic diarrhea of infant mice virus, ectromelia virus, Sendai virus, pneumonia virus of mice, Theiler murine encephalomyelitis virus, reovirus, lymphocytic choriomeningitis virus, mouse adenovirus, polyomavirus, *Mycoplasma pulmonis*, and pinworms. Housing was in a SPF barrier facility with a 12-hour dark/light cycle. Prior to experimental use, mice were acclimated for at least 6 days in ventilated microisolation cages in a temperature-controlled room (22.2°C ± 1.1°C) with relative humidity of 60% ± 10%. Mice had ad libitum access to food (Laboratory Rodent Diet 5001, PMI Lab Diet, St Louis, MO) and water.

### Study design

After an initial acclimation period at standard temperatures, mice were randomly placed in static cages (3–5 mice/cage) in specialized climate chambers (Powerscientific, Pipersville, PA). Chamber temperatures were set to either 22°C or 30°C with relative humidity set to 30%. After 7 days, groups of mice (n = 10/group) were euthanized without further manipulations to establish the effects of ambient temperature on select markers of inflammation including cell counts and cytokine concentrations in plasma and peritoneal lavage fluid. Spleen cells were harvested for total counts and *in vitro* stimulation. Additional groups of mice exposed to either 22°C or 30°C underwent cecal ligation and puncture surgery to induce polymicrobial peritonitis, then returned to their assigned housing temperature. For survival studies, septic mice were observed for 7 days after surgery. To evaluate inflammatory responses, septic mice were euthanized 6 hours after surgery for examination of systemic (blood) and local (peritoneal lavage) markers of inflammation.

### Cecal Ligation and Puncture (CLP)

After 7 days of housing in temperature chambers, mice (n = 10/group) were anesthetized with isoflurane (Vetone, Boise, ID). Through a ventral laparotomy, the cecum was exposed for a 50% ligation and two punctures with a 26-gauge needle using previously described methods to induce sepsis [20]. The abdominal musculature was sutured closed followed by closure of the skin with tissue glue. Mice received pre-emptive buprenorphine-HCl (0.05mg/kg, subcutaneous) diluted in 1.0 ml saline and subsequent doses in 0.1ml saline every 12 h for 48 hours post-operatively. Mice received heat support during anesthetic recovery. Post-operatively, septic mice were returned to the same housing temperature as their pre-operative acclimation period and were observed closely. Loss of righting reflex was used as a humane endpoint. The experimental reproducibility was supported by performing CLP in two independent trials and then combining the results.

### Temperature measurement

Mice acclimated for 7 days underwent CLP and transponders (IPTT-300; BioMedic Data systems, Inc, Seaford, DE) were inserted into the peritoneal cavity to allow 72 hours of temperature readings. To compensate for high, early sepsis mortality, sample size was increased (n = 15–17/group). Temperature readings were obtained with a corresponding handheld reader.

### Euthanasia and sample harvest

Mice were deeply anesthetized with isoflurane, and blood was collected from the retro-orbital sinus (500 μL into 50 μL of 169 mmol EDTA), followed by cervical dislocation. Peritoneal lavage fluid (PLF) was collected by injecting 10 ml Hanks Balanced Salt Solution (Invitrogen, Grand Island, NY) containing 1:100 heparin sodium (5000 USP U/mL; Abraxis, Schaumberg, IL) into the abdomen and retrieving fluid.

### Sample analysis

After collection, samples were placed in tubes labeled with impartial numbers to avoid bias during processing. The numbers were linked to a master list identifying individual mice and treatment groups.

#### Cell counts.

A 20-μL aliquot of blood was used for automated CBC analysis (Hemavet, Drew Scientific, Miami, FL). The remaining blood and PLF were centrifuged (2000 x g, 5 min, and 600 x g, 5 min, respectively) and supernatants stored at −80 °C for later cytokine analysis. The cell pellets from PLF samples were resuspended in 200 μL RPMI 1640 (Invitrogen) containing 0.1% heat-inactivated fetal bovine serum (Invitrogen). Cells were counted using a hemocytometer (Hausser Scientific, Horsham, PA). Slides were loaded with 1 x 10^5^ cells by centrifugation (109 x g, 5 min), and stained with Diff-Quick (Baxter, Detroit, MI). Differential cell counts (300 cells) were performed under light microscopy.

#### Bacterial Cultures.

Colony forming units (CFUs) in PLF were determined by serial dilution in PBS (0, 1:10, 1:100, and 1:1000) and direct plating on 5% sheep blood agar (Remel, Lenexa, KS). Plates were incubated overnight (37°C) and colonies counted on plates yielding 30–300 colonies.

#### Splenocyte culture and stimulation.

Spleens harvested from mice after a 7-day acclimation period were macerated, combined with 10 mL 1X PBS (ThermoFisher, Waltham, MA) and passed through 100 μM filters (Thermo Fisher). Samples were centrifuged at 800 x g for 5 min and red blood cells were lysed with Red Blood Cell Lysis Buffer (ThermoFisher). The cells were re-suspended in 1X PBS, diluted 1:2 with Trypan blue (ThermoFisher), and counted with a hemacytometer (Hausser Scientific, Horsham, PA). Cells were resuspended at 5x10^6^ cells/ml and plated in 24 well plates (Becton Dickinson, Franklin Lakes, NJ) in mouse lymphocyte medium consisting of 500mL RPMI 1640, 60mL heat inactivated bovine serum, 6mL Penicillin-Streptomycin Glutamine solution (Thermo Fisher), 6mL 100mM Sodium Pyruvate and 0.6mL 50mM 2-Mercaptoethanol (2-ME) (MiilporeSigma, St. Louis). Cells were stimulated by adding 5ug/ml of ConA (MilliporeSigma) to culture media or left unstimulated as controls. Cells were harvested from individual wells at 24, 48, 72, and 168 hours of culture and supernatants were stored at -80°C.

#### Cytokine measurements.

Cytokines were measured in plasma (1:10 dilution), peritoneal lavage fluid (1:2 dilution), and the supernatant from splenocyte cultures (1:10 dilution) using sandwich ELISA kits for CXCL1/KC, IL-6, CXCL2/MIP-2, and IFN-γ by DuoSet ELISA kits (R&D systems, Minneapolis, MN) according to manufacturer’s instructions. Absorbance was read at 450nm on a plate reader (Biotek, Winooski, VT). The data were analyzed using KC4 software (BioTek, Winooski, VT). The lower limit of detection was determined to be 15.8 pg/ml for IL-6 or CXCL1/KC and 31.0 pg/ml for CXCL2/MIP-2 or INF-γ.

### Statistics

All analyses were performed using Prism 10 for Windows (Graphpad Software, LaJolla, CA). For the survival study, Kaplan-Meier survival curves were calculated for each group, and differences between groups measured with log-rank tests. Data from endpoint measurements were tested for normality with a Shapiro-Wilk test and expressed as mean and standard deviation. Based on normality, comparisons between two groups were made with two-tailed Student’s t-test or Mann Whitney U. Comparisons of inflammatory responses over time and between temperature groups were performed with 2-way ANOVA and post-hoc Sidak’s multiple comparisons test. Comparisons of *in vitro* proliferation between stimulated and unstimulated temperature groups over time were performed with 3-way ANOVA and Tukey’s multiple comparisons for post-hoc testing. Power analyses (PS: Power and Sample Size Calculations, version 3.1.6) employing cell and cytokine data from our previous *in vivo* sepsis studies [21] indicated a sample size of 10/group would be sufficient for a Type I error probability of 0.05 and a power of 0.80.

## Results

### Thermoneutral acclimation and housing decreases sepsis survival.

**Fig 1:**
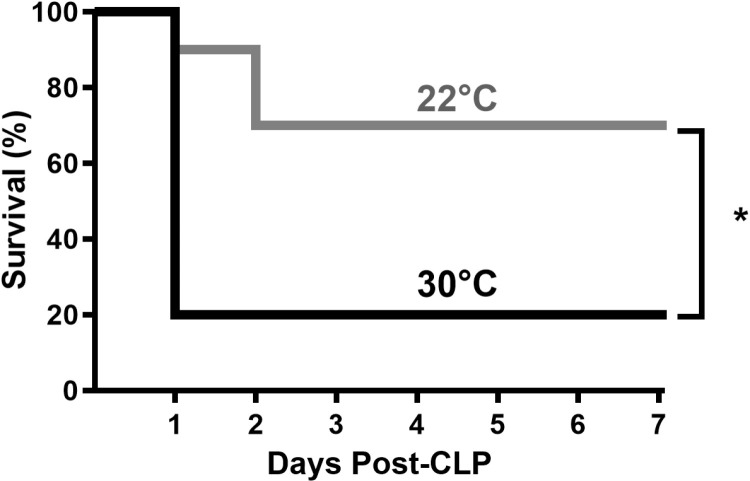
Thermoneutrality decreased sepsis survival. Mice were acclimated at either 22°C or 30°C for seven days and returned to their respective housing temperatures for observation up to 7 days after CLP. n = 10 mice/group, * = p < 0.05.

**Fig 2:**
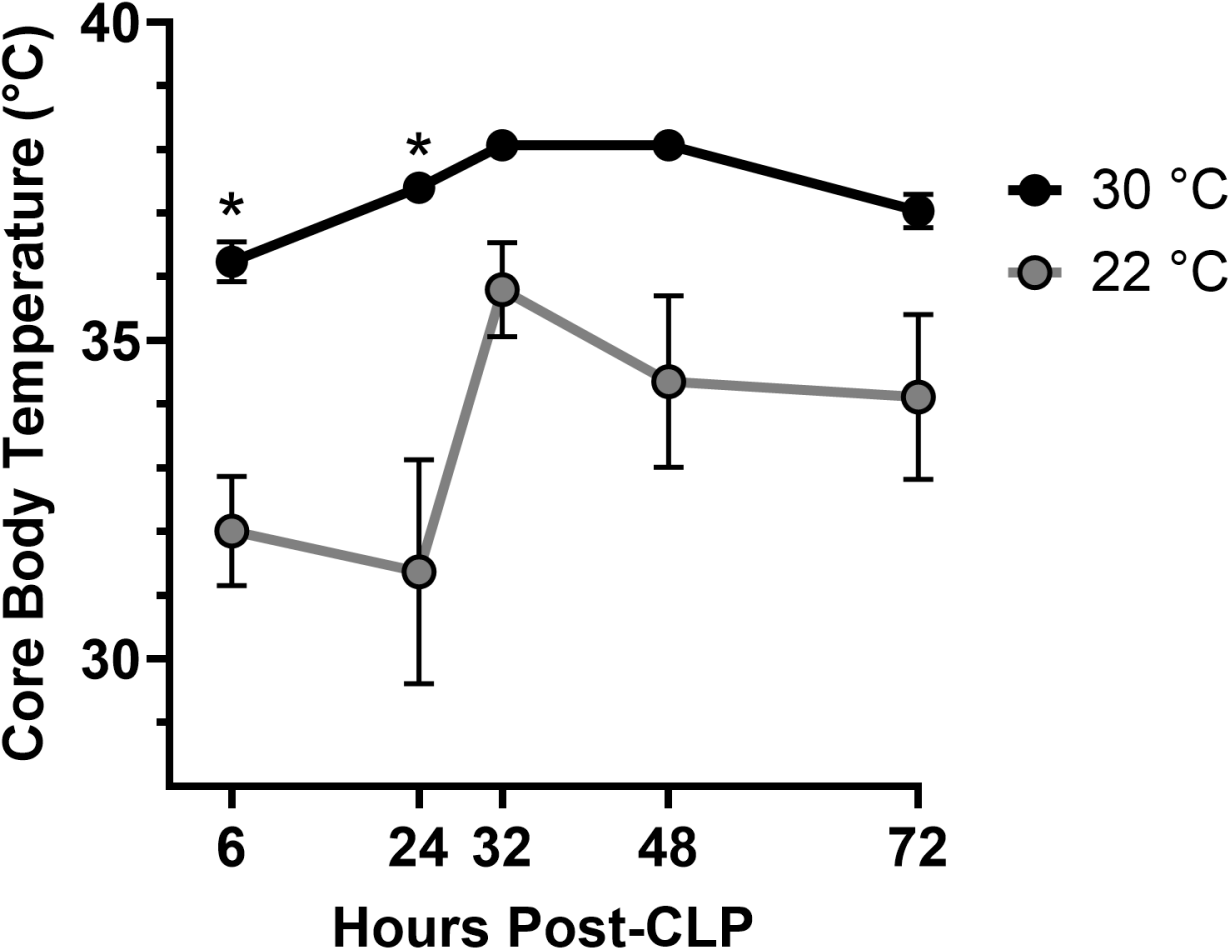
Thermoneutrality altered thermoregulation during sepsis. Mice acclimated at 22°C or 30°C for 7 days prior to CLP surgery and thermistor implant. Post-operative readings were obtained after return to respective ambient housing conditions. Data expressed as mean ± SD. n = 15-17 mice/group. * = p < 0.05.

Septic mice acclimated and housed at 30°C experienced significantly higher mortality than the mice at 22°C (80% vs 30%, respectively; p = 0.012; Fig 1). The 30°C group had a median survival of 24 hours whereas > 50% of the 22°C group survived for the seven-day observation period. The low survival in the 30°C group occurred despite early maintenance of core body temperatures (Fig 2). The mean body temperature over the first 24 hours was statistically higher (p < 0.0001) for the 30°C group (36.4 ± 1.3°C; range 33.4–38.4°C) as compared to the hypothermic 22°C group (31.7 ± 4.7°C; range 22.7–37.1°C).

Based on survival results, subsequent studies comparing the systemic and local responses were performed at six hours post-CLP. This timepoint was selected because it reliably preceded the time point associated with high mortality in survival studies.

### Thermoneutrality influenced systemic inflammation

#### Complete blood counts.

**Fig 3:**
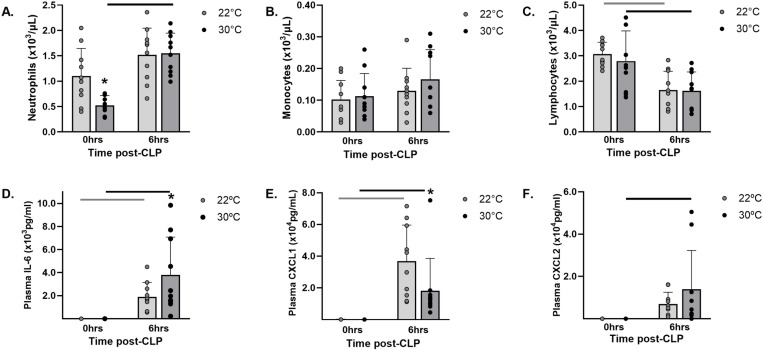
Thermoneutrality altered systemic inflammation before and after CLP. Mice were acclimated in either 22°C or 30°C for seven days. Blood was obtained on separate groups of mice either before (0hrs) or 6 hours after CLP. Whole blood cell counts including (A) neutrophils, (B) monocytes, and (C) lymphocytes and plasma cytokine levels including (D) IL-6, (E) CXCL1/KC, and (F) CXCL2/MIP-2 were obtained. Data expressed as mean ± SD. * = p < 0.05 between temperature groups at the same time point. Bars indicate p < 0.05 within a temperature group over time. n = 10/group.

Prior to CLP, the mice exposed to 30°C for 7 days had significantly lower circulating neutrophil counts (p = 0.011, Fig 3.A) as compared to the mice at 22°C (0.52 ± 0.20 vs. 1.10 ± 0.54 x10^3^/μL). Within 6 hours of CLP, neutrophil counts increased significantly (p < 0.001) in the mice exposed to 30°C (Fig 3.A) and reached levels comparable to the mice in 22°C (1.55 ± 0.40 vs. 1.52 ± 0.53 x10^3^/μL). Ambient temperature did not significantly impact the monocyte counts before or after CLP (Fig 3.B). Although the lymphocyte counts declined in the 30°C (p = 0.006) and 22°C (p = 0.001) after CLP, consistent with a septic response, there were no differences based on housing temperature (Fig 3.C).

#### Plasma cytokines.

After 7 days of acclimation and prior to CLP, plasma levels of IL-6 (Fig 3.D) were below the lower limit of detection for the ELISA. In response to sepsis, plasma IL-6 increased significantly in both temperature groups and were significantly higher (p = 0.04) in the 30°C group (3.8 ± 3.3 x10^3^ pg/mL) compared to the 22°C group (1.9 ± 1.3 x10^3^ pg/mL). Prior to CLP, plasma levels of the chemokine CXCL1/KC (Fig 3.E) were not significantly different between the groups acclimated at 30°C (83.4 ± 31.6 pg/mL) versus 22°C (193.3 ± 124.4 pg/mL) and were below the assay limit of detection for CXCL2/MIP-2α (Fig 3.F). After CLP, the plasma levels of CXCL1/KC (Fig 3E) increased in both groups but were significantly lower (p = 0.02) in the 30°C (1.8 ± 2.0 x10^4^ pg/mL) compared to the 22°C group (3.7 ± 2.3 x10^4^ pg/mL). The mean plasma levels of CXCL2/MIP-2α (Fig 3.F) rose in both groups after CLP but were not significantly different (p = 0.21) between the 30°C (13.9 ± 18.3 x 10^3^ pg/mL) and 22°C (6.9 ± 5.6 x10^3^ pg/mL) groups. Plasma levels of IFN-γ were below the lower limit of assay detection prior to and 6 hours after CLP.

### Thermoneutrality influenced local inflammation

#### Peritoneal cell counts.

**Fig 4:**
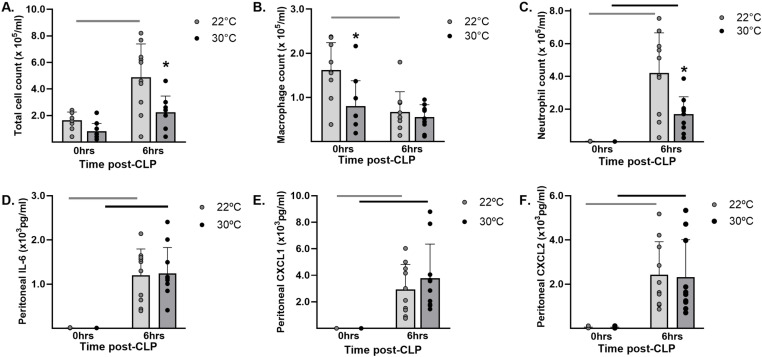
Thermoneutral temperatures altered peritoneal phagocyte counts. Mice were acclimated in either 22°C or 30°C for seven days. Peritoneal lavage fluid was obtained on separate groups of mice either before (0 hrs) or after CLP to induce sepsis (6hrs). Hemocytometer counts of cells, including (A) total cells, (B) macrophages, and (C) neutrophils and lavage fluid cytokines levels, including (D) IL-6, (E) CXCL1, and (F) CXCL2 were obtained. Data expressed as mean ± SD. * = p < 0.05 between temperature groups at the same time point. Bars indicate p < 0.05 within a temperature group over time. n = 10 mice/group.

After 7 days of temperature acclimation, peritoneal cell counts (Fig 4.A) consisted primarily of macrophages (Fig 4.B) present in significantly lower numbers (p = 0.002) in the 30°C group (0.80 ± 0.57 x 10^5^/μL) as compared to the 22°C group (1.62 ± 0.62 x 10^5^/μL). Peritoneal neutrophil counts were not statistically different between the 30°C (0.013 ± 0.012 x10^5^/μL) and the 22°C group (0.018 ± 0.015 x10^5^/μL) prior to CLP (Fig 4.C). After CLP, total peritoneal cell counts (Fig 4.A) were significantly lower (p = 0.0005) in the 30°C group (2.24 ± 1.22 x10^5^/μL) compared to the 22°C group (4.89 ± 2.5 x10^5^/μL), due to a less robust influx of neutrophils (1.68 ± 1.07 vs. 4.20 ± 2.46 x10^5^/μL, respectively; p = 0.0003: Fig 4.C).

#### Peritoneal cytokine levels.

Peritoneal lavage fluid levels of IL-6 (Fig 4.D) were below the limit of assay detection after 7 days of acclimation. The levels rose significantly after CLP within both the 30°C (1.2 ± 0.6 x10^3^ pg/mL) and 22°C groups (1.2 ± 0.6 x10^3^ pg/mL) but were not statistically different between the groups. Peritoneal lavage fluid levels for the chemokines CXCL1/KC (Fig 4E), and CXCL2/MIP-2α (Fig 4F) were below the lower limit of assay detection in mice after 7 days of acclimation. After CLP, the levels of CXCL1/KC demonstrated significant increases within the 30°C and 20°C groups (3.9 ± 2.8 x10^3^ pg/mL; p < 0.0001 and 2.9 ± 1.9 x10^3^ pg/mL; p = 0.0004, respectively) as did the levels of CXCL2/MIP-2α (2.3 ± 1.7 x10^3^ pg/mL; p < 0.0001 and 2.4 ± 1.5 x10^3^ pg/mL; p = 0.0001, respectively). However, chemokine levels demonstrated no differences between the temperature groups. The peritoneal levels of IFN-γ were below the limit of assay detection before and after CLP.

#### Bacterial burden.

**Fig 5:**
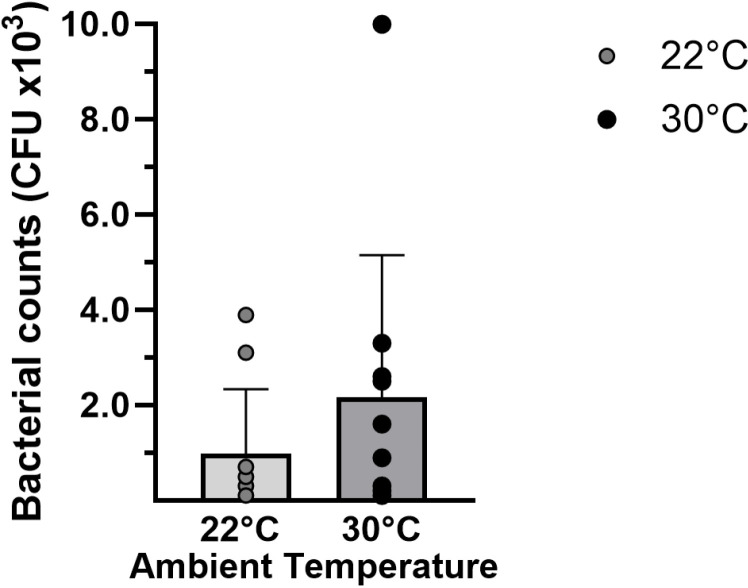
Peritoneal bacterial burden after CLP. Mice acclimated at either 22°C or 30°C for seven days underwent CLP. At six hours, peritoneal lavage fluid was collected and cultured overnight for bacterial counts. Data expressed as mean ± SD. n = 10 mice/group.

Overnight *in vitro* culture of peritoneal lavage fluid produced a mean bacterial count from the 30°C group that was 2-fold higher than from the 22°C group (respectively, 2.2 ± 0.9 vs. 1.0 ± 0.4 x10^3^ CFU) at 6 hours post-CLP; however, the difference was not significant (p = 0.54, Fig 5). Control peritoneal lavage fluid obtained from non-septic mice acclimated at either temperature yielded no bacterial growth.

### Splenocyte responses

**Fig 6:**
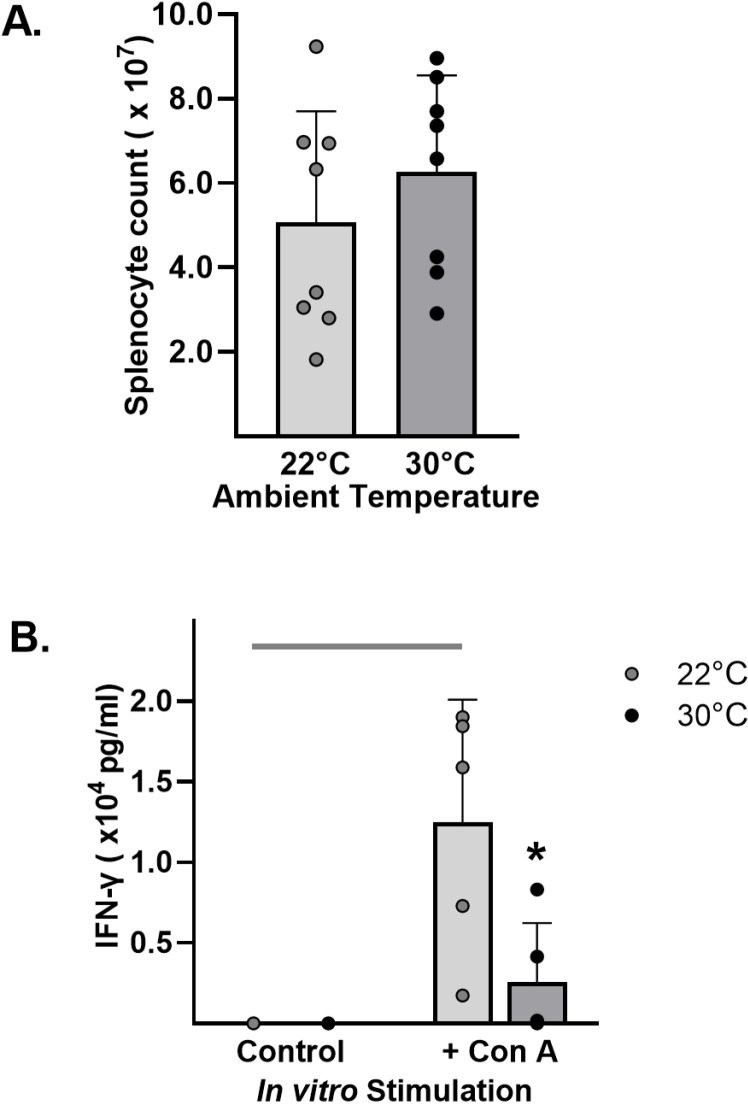
Thermoneutrality reduced i*n vitro* IFN- **γ** **release by splenocytes.** Mice were housed at either 22°C or 30°C for 7 days. Splenocytes were harvested and counted: (A) Total splenocyte counts before culture. Splenocytes were plated with or without Concanavalin A. After culture for 24 hours, supernatants were harvested for ELISA to determine (B) IFN-γ concentrations. Data expressed as mean ± SD. n = 5 mice/group. * = p < 0.050.

The absolute splenocyte counts from mice acclimated at either 30°C or 22°C (Fig 6.A) were not significantly different (5.07 ± 2.64 vs. 6.27 ± 2.29 x10^7^pg/mL, respectively; p = 0.35). After 24 hours of culture with or without stimulation with concanavalin A (Fig 6.B), the concentration of IFN-γ produced by splenic cells from mice acclimated at 30°C was significantly lower compared to mice acclimated at 22°C (0.38 ± 0.68 vs 2.55 ± 0.76 x10^4^ pg/mL, respectively, p = 0.004).

## Discussion

The study presented here demonstrated that acclimation and post-operative housing at a thermoneutral temperature (30°C) decreased survival after CLP when compared to standard housing temperature (22°C). Similarly, lower survival rates were seen with higher housing temperatures in rodent models after bacterial infection [14,18,22], endotoxin administration [14], and CLP [23]. Studies of seasonal variations in murine sepsis models (CLP and colon ascendens stent peritonitis) showed decreased survival in summer compared to winter, in the presence of mildly higher environmental temperature and humidity even within the regulated, standard ranges [24,25]. In contrast, other murine studies have shown increased survival after bacterial inoculation when mice were housed in thermoneutral temperatures as compared to standard temperatures [16,17,26–28]. Our previous study using a comparable CLP model showed a significant increase in survival (78% versus 40%) when mice were housed post-operatively at 30°C compared to 22°C [20]. Several factors could contribute to differences in the results between all these studies including type and severity of the insult [18] as well as strain and sex differences of mice. However, comparing this study with our previous work [20], the one remarkable difference was the inclusion of thermoneutral temperatures during the acclimation period prior to induction of sepsis. We theorize that the temperature history prior to bacterial exposure promoted several changes in innate immunity that significantly impacted survival.

The systemic inflammatory response after CLP was altered in the mice exposed to thermoneutral temperatures. Although initially depressed in the 30°C group, blood neutrophil counts demonstrated a significant rebound, suggesting a robust inflammatory response. This was substantiated by significantly increased plasma IL-6, a sensitive indicator of inflammatory status and an endogenous pyrogen [29]. Heightened systemic inflammation has been linked to early deaths in clinical cases and plasma IL-6 has been used as a prognostic tool in septic patients [30,31]. Similarly, high IL-6 levels at 6 hours post-surgery are predictive of mortality in the murine CLP model [32], suggesting an association between early inflammation and reduced survival with thermoneutrality in our study. Of note, systemic levels of IFN-γ were not detectable at the 6-hour time point; however, *in vitro* stimulation showed production of IFN-γ from splenocytes may be impaired in mice acclimated at 30°C. Others have shown, in a model of *K. pneumoniae* peritonitis, that thermoneutral housing did not alter peak plasma levels of IFN-γ but temporally delayed the peak by several hours [26]. Since a shift toward Th2 cytokines is a hallmark of poor sepsis outcomes [33], it is possible that an impaired or delayed IFN-γ response also could have contributed to the early mortality seen under thermoneutral conditions in our study. Experimental studies have attributed various patterns of increased [14,34–36], decreased [16,20,28], or similar [18,23] inflammation when mice were housed at thermoneutral compared to standard or slightly increased temperatures. The variability seen among studies suggest that factors in combination with the ambient temperature may influence the inflammatory response and determine outcome.

An assessment of factors local to the site of infection showed that acclimation at thermoneutral temperatures for seven days reduced peritoneal macrophage counts and lowered numbers of circulating neutrophils prior to CLP. The peritoneal recruitment of neutrophils in response to CLP was also significantly reduced or delayed in the 30°C group, despite comparable levels of local chemokines in both groups. Together, these findings suggest that thermoneutral housing reduced the potential for immediate innate responses at the local site of infection. Previously, we showed no differences in local cell counts by 12 hours after CLP when mice acclimated at standard temperatures were exposed to different post-operative housing temperatures [20]. However, other animal models have shown that acclimation and post-insult housing of rodents at thermoneutral temperatures may reduce local recruitment of immune cells, including lower total peritoneal inflammatory cell counts after infection with leishmania [37] and lower recruitment of neutrophils to cardiac tissue in response to LPS-induced shock [28]. The reduced recruitment of phagocytes in our study was not associated with a statistically significant increase in peritoneal bacterial burden in the 30°C group versus the 22°C group (2.16 ± 0.95 vs. 0.98 ± 0.43 x10^3^ CFU; p = 0.54). Although this may have been due to timing within our study, another study showed that ambient temperature itself did not directly influence local bacterial counts in a model of Klebsiella peritonitis [26] and others have shown that housing temperature affects the relative abundance of bacterial species, not absolute numbers [38]. Therefore, we cannot rule out the possibility that temperature-induced changes in the gut microbiome as well as inflammatory cell recruitment affected the survival outcomes in this polymicrobial model of sepsis.

Our current and previous [20] studies showed that thermoneutral housing eliminated the immediate hypothermia after CLP seen under standard housing temperatures. In this study, mice housed in thermoneutral conditions maintained body temperatures in the accepted normal range (~ 37°C) [39] and one developed a body temperature in the range considered fever in humans (>38.3°C) [40] These results are in keeping with other studies in which mice developed hypothermia after challenge with either LPS or infectious agents under standard temperature conditions but were normothermic or febrile under thermoneutral conditions [14–16,18]. Likewise, a recent study showed severe sepsis induced by CLP resulted in fever when rats were exposed to a thermoneutral environment [23]. Since septic clinical patients often become febrile during infection, our results could suggest the thermoneutral CLP model may provide better translation to the clinical scenario. However, in our current murine study and a CLP study in rats [23], higher body temperature was associated with greater mortality after CLP. This is counter to studies revealing higher mortality rates in septic patients with hypothermia [41,42] and to our previous studies in cold stressed mice. Although this might suggest the thermoneutral model may be less translatable, the results of this study illustrate the effects of distinct, conserved defense strategies, resistance versus tolerance, that stem from the need to balance the host’s existing energy stores during elimination of a pathogen [18,43,44]. In a study using either LPS or *E. coli* injections, mice in a stressed metabolic state induced by housing at 22°C shunted available energy away from homeostatic functions such as thermoregulation to mount an immune response [18]. Mediated by TLR4 signaling, this energetic trade-off resulted in a hypometabolic and hypothermic state that promoted protective tissue tolerance, resulting in enhanced survival. In contrast, well-fed mice in a basal metabolic state (30°C) maintained body temperatures while resisting the agent with an exuberant immune response that may be detrimental to unprotected tissues [18]. Therefore, temperature regulation and immune responses to infection may be determined by the metabolic state of the host. In fact, body temperature is dynamic and may follow several trajectories in septic patients [45], suggesting comprehensive preclinical studies may require more than one model to better represent the heterogenous array of responses in the patient population. A similar strategy using ambient temperature to produce models has been proposed for cancer studies [46].

Our study had some limitations. We chose to use a single strain which could influence the type of immune response and male mice that reduce the variability associated with estrus cycle [47]. The study focused on a finite set of immune markers at a very early time point to define factors associated with the differences in mortality. Longer term and broader studies will be needed to study chronic and recovery phases of sepsis. Finally, continuous rather than intermittent recording of body temperature would have detected transient hypothermia and/or fever and better defined the clinical implications of the models.

In conclusion, exposure to thermoneutral ambient temperature had significant effects on innate immune responses and outcomes of a murine CLP model. Notably, one week of thermoneutral acclimation reduced phagocyte numbers and limited the production of IFN-γ by stimulated splenocytes. In response to polymicrobial peritonitis, the thermoneutral environment preserved body temperature but resulted in a highly lethal model accompanied by enhanced systemic inflammation and reduced local recruitment of neutrophils. Further studies should elucidate the factors mediating the complex effects of ambient temperature and determine their relationship to the array of immune responses manifested by clinical patients with sepsis. In the meantime, these results underscore the importance of reporting ambient temperature as well as the length of the acclimation period at that temperature to enhance reproducibility in sepsis models.
